# Electrochemotherapy in Spine Metastases: A Case Series Focused on Technical Aspects, Surgical Strategies and Results

**DOI:** 10.3390/diagnostics14090936

**Published:** 2024-04-30

**Authors:** Andrea Angelini, Alberto D’Amico, Stefania Paolilli, Riccardo Signori, Giovanni Baldin, Giuseppe Di Rubbo, Luca Denaro, Pietro Ruggieri

**Affiliations:** 1 Department of Orthopedics and Traumatology and Oncological Orthopedics, University of Padova, Via Giustiniani, 35128 Padova, Italy; stefania.paolilli@aopd.veneto.it (S.P.); riccardo.signori88@libero.it (R.S.); giovannibaldin@gmail.com (G.B.); giuseppe.dirubbomd@gmail.com (G.D.R.); pietro.ruggieri@unipd.it (P.R.); 2 Academic Neurosurgery Department of Neurosciences, University of Padova, 35128 Padova, Italy; alberto.damico@unipd.it (A.D.); luca.denaro@unipd.it (L.D.)

**Keywords:** electrochemotherapy, spine tumor, metastasis, bone disease

## Abstract

Metastases are complications of primary tumors due to prolonged cancer survival and have become an important issue for oncological patients and the most frequent cause of death and disability. Bone metastases occur at a later stage of cancer disease, and the spine is the most frequent site. To date, the aim of the treatment of metastases remains to be the control of disease and provide a satisfactory quality of life. The decision making of treatment is influenced by several factors such as the status of the primary disease, the number of metastases, site involvement, and the performance status of the patients. For this reason, the treatment of metastases is challenging and undergoes constant development. Therefore, alternative techniques with respect to surgery, which is the first option but not always practicable, and radiochemotherapy are attractive. Lately, electrochemotherapy has emerged as an innovative method for treating various primary and metastatic solid tumors, showing promising outcomes in terms of inducing tumor tissue necrosis and alleviating symptoms. This technique uses electric pulses to increase the uptake of chemotherapy by tumor cells. Despite the initial enthusiasm and good results in the treatment of bone tumors, relatively few papers have described its use in spine metastases. Therefore, we conducted a systemic review of this intriguing topic while also reporting our experience in the use of electrochemotherapy for the treatment of spine metastases.

## 1. Introduction

Bone is the third most common site of metastases following the lung and liver, and the spine is the most common site for bone metastasis [1]. The highest incidence of spine metastases occurs from 40 to 65 years of age [2]. The thoracic spine is the most common site of involvement (70%), followed by the lumbar spine (20%), the cervical spine, and the sacrum [3]. The vertebral body is involved in 80% of cases, with posterior elements being affected in 20%, and most metastases are osteolytic (95%) [4,5]. The paramount purpose of spine metastasis treatment is to correct mechanical instability, resolve eventual neurological compression, and improve quality of life [6]. 

Treatment in spine metastases has changed over the years, and several techniques are described in the literature [6,7,8,9]. In this scenario, recently, electrochemotherapy (ECT) has been demonstrated to be an efficient treatment for spine metastases [10]. This technique combines systemic chemotherapy, usually bleomycin, and electric pulses delivered locally at the tumor site. The intriguing mechanism of this technique is characterized by the temporary permeabilization of cell membranes of tumoral tissue that allow bleomycin to diffuse inside cells and increase its toxicity. It is also used in different settings such as benign tumors of the spine [11]. Several studies have reported the efficacy and feasibility of this technique in the treatment of bone metastasis [12], but papers about this topic focused on spine metastasis are sparse, and to date, a standardized protocol has not been described. Therefore, a systematic review of the literature on the use of ECT in spine metastasis was conducted. Additionally, we report our initial experience in the treatment of spine metastases with ECT.

## 2. Material and Methods

*Systematic review of the literature*: Given the sparse evidence about ECT in metastatic spine disease, a systematic literature review was performed according to Preferred Reporting Items for Systematic Reviews and Meta-Analyses (PRISMA) guidelines. The protocol of this review was not registered on prospective registers; however, the systematic review was conducted as follows: A literature search was performed on the PubMed database in October 2023. PubMed was queried without backward limits with the following terms: ((spine) AND (metastasis)) AND ((ECT); (ECT) AND (spine)); ((ECT) and (metastasis)). Reference lists of all publications were also screened for missed results. Exclusion criteria were inaccessibility to full text, non-English and non-Italian language, studies not pertinent to ECT in spine disease by “title/abstract”, and studies lacking relevant clinical data (clinical presentation, demographics, surgical procedure, and radiological findings). Review articles were also excluded.

Data extracted from each study included article type, class of evidence if reported, the spine side involved, the type of primary tumor, ECT technique, and functional outcome at the last follow-up. Clinical outcome was evaluated as pain relief, measured by a VAS score ranging from 0 (no pain) to 10 (worst pain ever) and the American Spinal Injury Association (ASIA) impairment scale [13].

*Our protocol and technical aspects*: While under general anesthesia, the patient is positioned prone, followed by the execution of a posterior surgical approach involving laminectomy at the targeted level. This approach ensures sufficient exposure of the pedicle area. Particular attention is paid to avoiding damage to nerve roots and the dural sac. Two stainless steel needle electrodes, each measuring 1.8 mm in diameter with sharpened tips, are then inserted into both pedicles of the affected vertebra, with an additional set of four electrodes placed in the pedicles of the adjacent proximal and distal vertebrae. These placements are guided by fluoroscopy and/or neuronavigation techniques. The electrodes are linked to the independently regulated generator outputs of the Cliniporator Vitae (IGEA, Carpi, Italy). This Cliniporator Vitae unit functions as a pulse generator, featuring six electrically isolated outputs, each capable of delivering up to 3000 V with a maximum current of 50 A. It administers eight rectangular electrical pulses, each lasting 100 ms with a rise time of 1 ms, at a pulse repetition rate of 4 Hz [14]. Systemic bleomycin (Bristol-Myers Squibb, Princeton, NJ, USA) is administered intravenously (i.v.) via push injection, typically over a period of 30 to 45 s, at a dosage of 26 mg. This injection is conducted following electrode connections eight minutes before the delivery of electrical pulses. The administration of bleomycin adheres to the ESOPE protocol, which specifies doses of 10 or 15 mg/m^2^ [15].

Following an 8 min interval, pulse delivery is initiated, with continuous monitoring to ensure the efficacy of the applied electrical field, set at 1.5 A. Both current and voltage levels are meticulously measured and recorded with a precision exceeding 3%, facilitating precise control over pulse delivery and enabling thorough evaluation post-treatment. Throughout the administration of electrical pulses, the patient undergoes partial curarization.

## 3. Results

The literature search provided the following results: ((spine) AND (metastasis)) AND ((ECT) 3 results; (ECT) AND (spine)) 5 results; ((ECT) and (metastasis)) 137 results. Through this literature search, we retrieved a total of 145 papers. 

**Figure 1:**
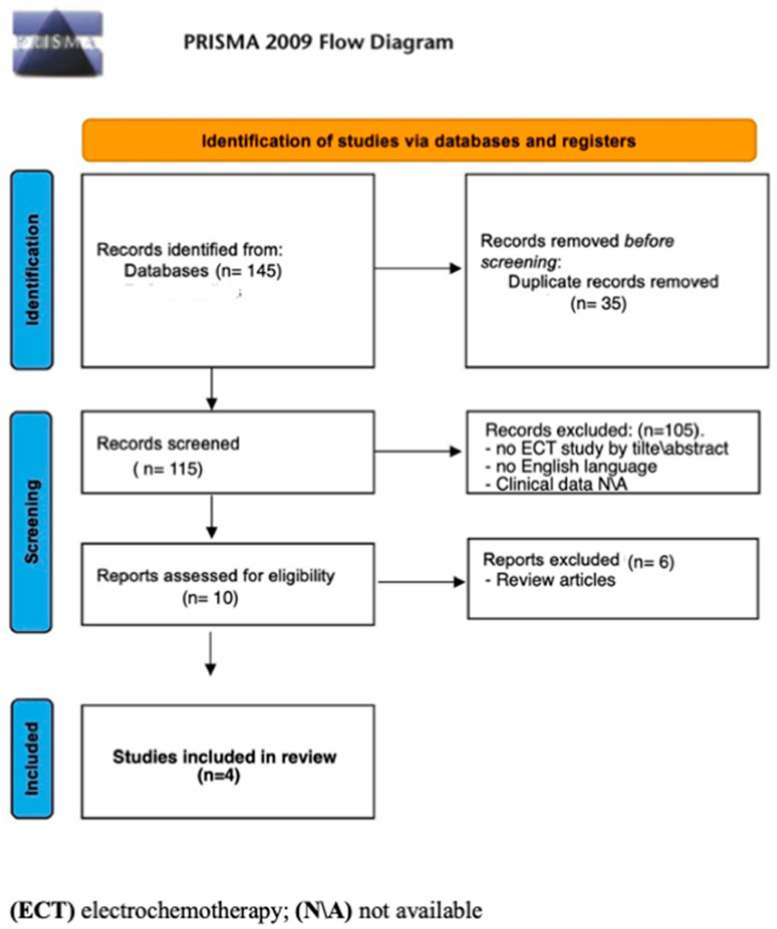
PRISMA flow diagram of the literature review.

After careful screening following the PRISMA flowchart and considering the purpose of this systematic review and the exclusion criteria, only four studies [12,16,17,18] were retrieved and assessed for eligibility (Figure 1; Table 1).

## 4. Case Series

**Figure 2:**
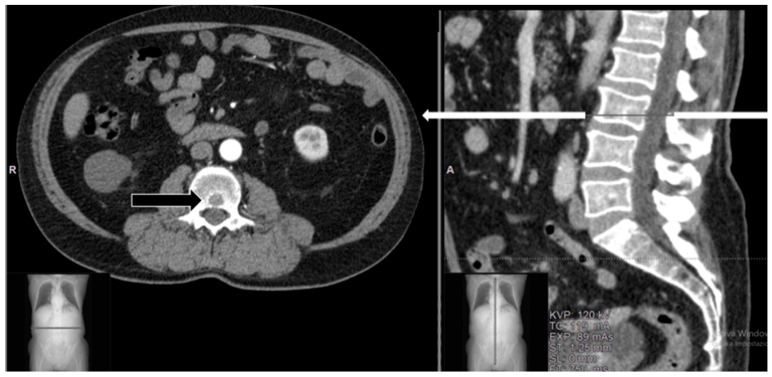
Case 1. A 75-year-old female with small lytic metastasis from high-grade myxofibrosarcoma in the L3 vertebra. The lesion was confined to the vertebral body (black arrow) without the involvement of the posterior vertebral wall. White arrow in sagittal view corresponds to the axial level.

A 75-year-old female Caucasian patient with no history of oncologic disease presented with swelling on the anterior surface of the left leg. Imaging studies revealed a soft tissue suprafascial mass diagnosed as high-grade myxofibrosarcoma (G3 at FNCLCC classification) upon histopathologic evaluation of percutaneous tru-cut biopsy. Subsequently, the patient underwent surgical excision and soft-tissue reconstruction with a myocutaneous flap and cutaneous Thiersch graft. Local radiotherapy was completed with a dosage of 36 Gy within 6 months postoperatively. Six months after the completion of radiotherapy, the patient presented with suspected micronodularity in both lungs and a small lytic lesion in the L3 vertebra (Figure 2), not associated with low back pain (VAS 1). 

**Figure 3:**
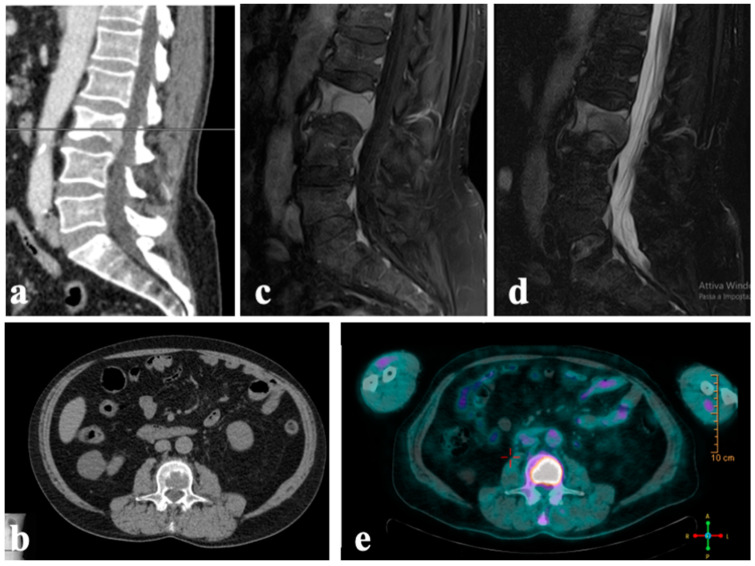
Case 1 at 3 months of follow-up: (**a**) Sagittal and (**b**) corresponding axial CT scans show a lytic lesion in the L3 vertebra with posterior erosion of the cortex, involving the spinal canal. (**c**) Sagittal T1-weighted MR imaging with contrast medium shows a homogeneous lesion at the L3 level with mixed hyperintense signal, whereas in (**d**) sagittal T2-weighted MRI, the lesion appears hypointense. (**e**) FDG-PET/CT on axial view shows the intense focal area of 18-FDG activity in the L3 vertebral body.

A decision was made to conduct a short follow-up re-evaluation, which was agreed upon by the patient. Three months later, a new CT scan revealed that the lesion in the L3 vertebra had increased in volume (Figure 3a,b), and MR imaging showed partial compression of the medullary canal (Figure 3c,d). FDG-PET/CT was performed, showing increased uptake (SUV 12) (Figure 3e). The patient reported a significant worsening of pain, with pain radiating to the left thigh, but there were no ongoing peripheral neurological deficits (ASIA scale E). 

Afterward, the patient was referred to our oncologic spine surgery department, where a percutaneous biopsy of the L3 vertebral body was performed. The results of histopathological examination and immunohistochemistry indicated metastatic high-grade myxofibrosarcoma. Considering the patient’s oncologic scores, which suggested the need for locoregional treatment at the L3 level, surgical planning began. Notably, there were no signs of spine instability or significant neurological deficits detected at that time, but the growth of the lesion was concerning. 

**Figure 4:**
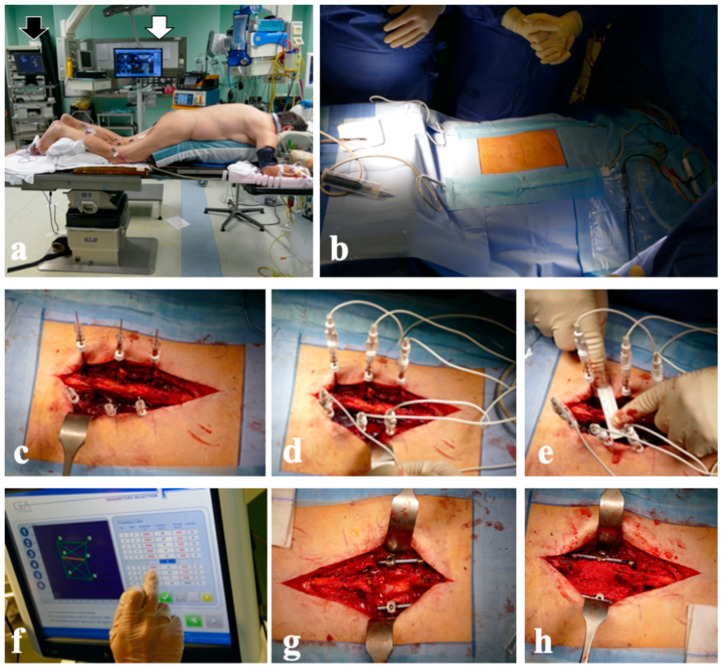
Intraoperative pictures: (**a**) positioning of the patient in the operating room. The devices for intraoperative navigation (white arrow) and the electrodes for intraoperative evoked potentials (black arrow) are visible; (**b**) sterile field; (**c**) precise alignment of needle electrodes surrounding the metastatic lesion was attained under the guidance of navigation and fluoroscopy; (**d**) the electrodes were connected to the pulse generator; (**e**) the distance between each couple of electrodes was registered and (**f**) reported on the device; (**g**) after ECT, electrodes were replaced by pedicled screws and laminectomy for spinal decompression was performed; (**h**) dura covered with bone graft.

Subsequently, a wide laminectomy procedure coupled with stabilization utilizing pedicled screws and ECT was proposed as a local/palliative treatment approach. This strategy aimed to slow down rapid disease advancement while minimizing the potential for intolerable morbidity associated with extensive surgical resection. The patient consented to this option after being fully informed. The surgery was performed with the patient positioned prone under the navigation and fluoroscopic guidance (Figure 4). 

**Figure 5:**
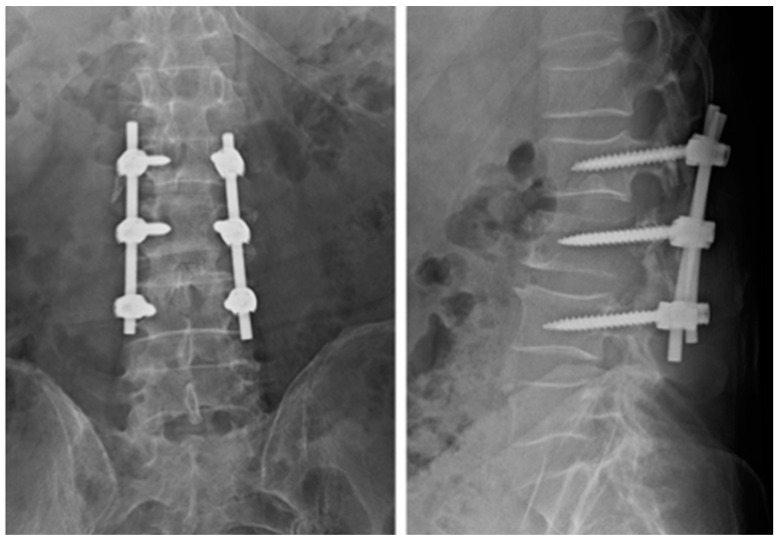
Case 1. Radiographs taken 3 months after surgery demonstrate no signs of implant loosening.

Follow-up assessments, comprising both clinical and radiological examinations, were scheduled at one- and four-week post-surgery, followed by three-month intervals thereafter. The evaluation of the patient’s reported outcomes using the Visual Analog Scale (VAS) revealed a statistically significant reduction from 10 to 2 (*p* < 0.001). However, no significant changes were noted in the patient’s neurological status, and there were no local or general side effects observed either immediately after waking or during postoperative follow-up. Imaging restaging conducted 3 months after surgery (Figure 5) demonstrated no signs of implant loosening, and the size of the tumor at the L3 vertebra remained stable. Unfortunately, the patient passed away at 4 months of follow-up due to respiratory failure caused by the progression of metastatic disease to the lungs. 

A 76-year-old female patient with a history of squamous cell carcinoma of skin in the lumbar area, which was surgically removed in November 2022 with clear surgical margins, and abdominal scleroatrophic lichen presented to our spinal surgery center with symptoms of spinal cord compression. These symptoms included a 2/5 strength deficit in the quadriceps femoris, psoas, and right adductor muscles; bilateral patellar and midplantar hyporeflexia; absence of clones; negative Babinski sign; and preserved sensitivity in the lower limbs.

**Figure 6:**
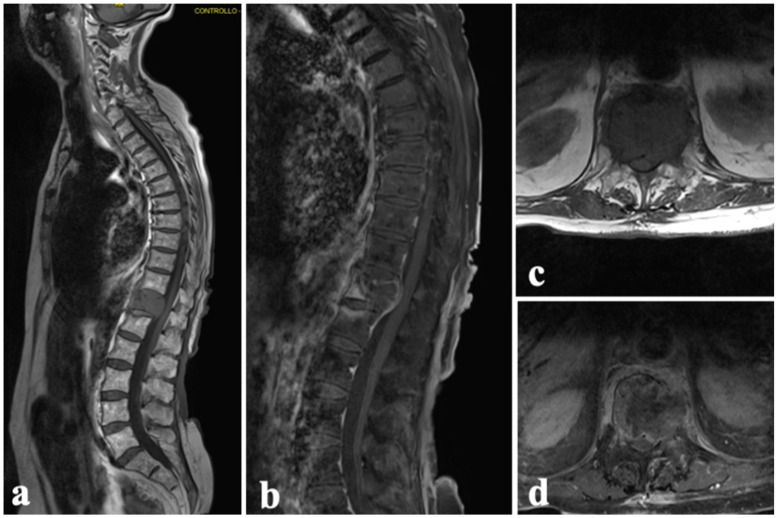
Case 2. A 76-year-old woman with T12 metastasis from squamous cell carcinoma: (**a**,**b**) preprocedural MRI in sagittal and (**c**,**d**) axial plane showing the complete involvement of anterior vertebral body and partial cord compression.

Upon instrumental examination (whole-spine MRI), an osteolytic lesion was observed on the vertebral soma of D12 (Figure 6), along with a pathological fracture and medullary compression. 

**Figure 7:**
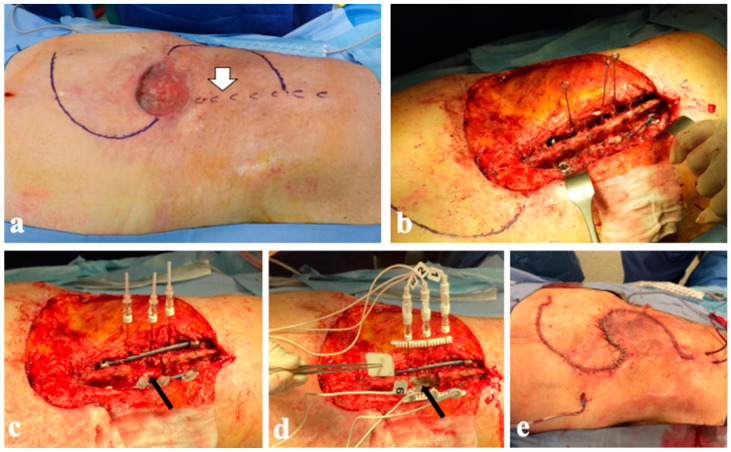
Case 2. The patient was previously treated with wide surgical removal of cutaneous squamous cell carcinoma of the dorsum. She developed a T12 bone metastasis (white arrow). Intraoperative pictures of subsequent surgical procedures with decompression, pedunculated flaps, and image-guided ECT of spine metastasis are shown: (**a**) soft-tissue defect, drawing of cutaneous incision correlated with T12 vertebra (white arrow); (**b**) spinal stabilization with the four furthest screws and Kirschner wire into other pedicles; (**c**) electrodes were inserted, and wide laminectomy was performed (black arrow); (**d**) electrodes were connected with pulse generator; (**e**) surgical field after spinal instrumentation and wound closure.

A needle biopsy was performed, yielding a diagnosis of malignant epithelial neoplasm with squamous differentiation, suggestive of distant metastasis. At the same time, the patient underwent radiotherapy (60 Gy within 3 months of the skin excision procedure). Three months later, imaging restaging revealed stationary disease, with minimal pain and worsening quality of life. For these reasons, a recommendation was made for strict follow-up and conservative treatment with a Camp C-35 brace. In the following month, clinical deterioration was noted, characterized by increased pain and a strength deficit of 4/5, with Babinski sign +/− and ASIA scale C. Oncological scoring systems (Karnofsky 50%, Tokuashi 11, Tomita 3) indicated a favorable prognosis, and following discussions with the patient, a surgical recommendation was made for spinal cord decompression at the D12 level, spinal stabilization from D11 to L2, ECT with bleomycin administered at the D12 vertebra, and plastic reconstruction of the skin defect (in the area of the previous excision of squamous cell carcinoma) using pedicled surgical flaps (Figure 7).

A histopathological examination of the vertebral lesion was performed concurrently with histological confirmation of keratinizing squamous carcinoma. The patient reported a significant reduction in pain (VAS 2) and the disappearance of peripheral neurological deficits (ASIA scale E). However, complications arose from the surgical wound, including the dehiscence of the distal third and the partial necrosis of the flap, which required further plastic revision surgery. During hospitalization, the patient developed a cerebellar stroke, resulting in progressive worsening of neurological imaging. Additionally, bilateral pleural effusion occurred, necessitating the placement of a pleural drain. At the 3-month follow-up, the patient experienced acute renal failure, ultimately leading to death.

A 73-year-old male patient with no history of tumor presented with swelling in the left ankle for about a year, with a notable increase in size in the last 3 months. An MRI of the left ankle showed a soft-tissue lesion in the distal third of the leg with no erosion of the bony cortex. A needle biopsy with histological evaluation was performed, resulting in the diagnosis of leiomyosarcoma G3 with areas of necrosis (30%) desmin +, caldesmon +, and smooth muscle actin +. A below-knee amputation was performed with histological confirmation of both tibial and fibular bone infiltration and wide margins.

**Figure 8:**
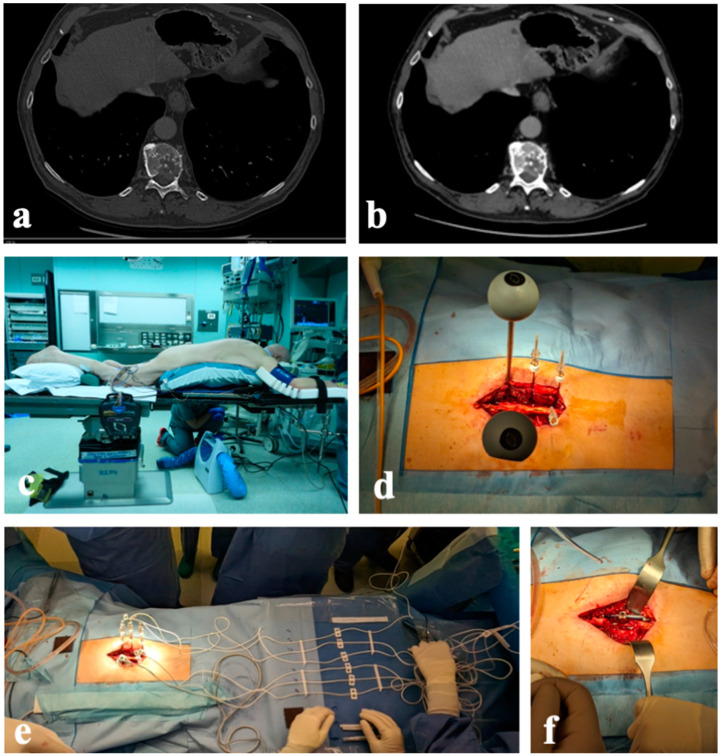
Case 3. A 73-year-old man with T11 metastasis from leiomyosarcoma grade 3: (**a**,**b**) axial CT scan shows the lytic lesion with spinal cord compression; (**c**) positioning of the patient; (**d**) insertion of the electrodes; (**e**) connection of the electrodes with the pulse generator; (**f**) definitive instrumentation and laminectomy.

During follow-up, a CT scan of the chest and abdomen for restaging showed multiple metastases in the pectoral region, left gluteal region, left ischiopubic branch, and the vertebral soma of D11, with the involvement of the posterior wall and partial spinal cord compression (Figure 8a,b). Given the effective pain control and the absence of neurological symptoms, conservative treatment with a spinal brace was proposed for the thoracic metastatic lesion, along with chemotherapy. During the first month, the patient experienced clinical worsening (ASIA scale D), reporting an increase in pain to VAS and a decline in oncological indices (Karnofsky 50%, Tokuashi 9, and Tomita 6). Therefore, surgical treatment was considered. Surgery consisted of spinal cord decompression in D11, spinal stabilization from D10 to L1, and ECT with bleomycin administered at the D11 vertebra (Figure 8c–f), with immediate pain relief (VAS 2) and neurologic restoration (ASIA scale E). The patient was alive with disease at the 6-month follow-up, with stable disease observed in the vertebral site.

## 5. Discussion

Undoubtedly, ECT is an important option to manage primary tumors and metastases [19]. Okino et al. [20,21] and Mir et al. [22] provided initial descriptions of a technique involving the local application of electric current to enhance the uptake of chemotherapy drugs, typically bleomycin, into tumor cells. This approach has proved promising regardless of tumor histology [23]. Recently, ECT has been used in more than 83 centers all over Europe [24], and studies have shown that it can also potentially be used in the treatment of bone tumors and bone metastases. To date, its use in clinical practice as a new option for metastasis management is increasing; however, while several studies have been conducted about this technique in the treatment of bone metastasis, few papers have described its use and results in spine metastases [12,16,17,18]. In fact, from our systematic review, only four articles described the use of ECT in spine metastases [12,16,17,18].

Spine metastases are the most common bone tumors and a frequent occurrence in the spine. Therefore, in managing this challenging issue, ECT would work together with medical therapy, surgery, and chemo- and radiotherapy to enhance the quality of life for these patients. The primary goal of treating patients with spinal metastases is palliative, focusing on improving pain control, which is often the foremost concern for these individuals, while also aiming to preserve spinal function and stability [25]. Traditionally, medical therapy, surgery, chemotherapy, and radiotherapy are valid options for managing oncological patients with a risk–benefit–cost ratio that is not always favorable [26]. For this reason, new strategies of treatment with low comorbidity are attractive. In this scenario, ECT is a new tissue-sparing treatment with selective destruction of tumor cells. In 2015, Gasbarrini et al. [12] were the first to report the use of ECT in the palliative treatment of spinal metastases. They described a case involving a 51-year-old female with melanoma metastasis in the L5 spine. This option was chosen based on oncological scores [27,28]. The electrodes were placed directly in the L5 vertebral body before decompression. The chemotherapy drug delivered was systemic bleomycin. The procedure was tolerated without side effects. Response assessment included percutaneous vertebral biopsy at 3 months (95% of tumor necrosis occurred) and PET/TC at 6 months. Then, the patient underwent radiotherapy for a suspected local relapse identified on the PET/TC scan. In 2018, Cindrič et al. [16] proposed a new way to deliver electrical pulses near tumor tissue, describing two cases of spine metastases treated by ECT with a transpedicular approach. The authors demonstrated nearly 100% coverage of tumor volume with the electric field delivered via a transpedicular approach [16]. Moreover, when the tumor was located in the anterior part of the vertebral body, no electroporation damage was observed on spinal cord tissue [16]. In this situation, the electrodes have more distance to the target, and a higher electrical pulse is needed to provide electroporation effects on tumor tissue. This is an important concept when treating spine metastases because the potential damage to the spinal cord and roots needs to be considered. Cornelis et al. [17] reported their experience of percutaneous image-guided ECT in two patients with metastatic epidural spinal cord compression from breast and lung cancer. Both had previously undergone chemotherapy, and radiotherapy was performed to relieve back pain with no good result. Thus, ECT was proposed as an alternative palliative treatment. A transpedicular technique was used, and electric pulses were delivered near tumor tissue by inserting electrodes in the affected vertebral body and at the above and below levels with a CT-guided procedure. Electric pulses were delivered 8 min after the intravenous administration of bleomycin. The treatment was well tolerated, and no side effects occurred. MRI confirmed the tumor’s local response, and a biopsy at 3 months of follow-up showed >95% necrosis of tumor tissue [17]. Recently, Deschamps et al. [18] reported the most numerous series of spine metastatic diseases treated with ECT and bleomycin. The authors reported a total of 40 patients with metastatic epidural spinal cord compression. All patients in this series received radiotherapy, and 12 patients had a previous surgery. ECT was performed with the percutaneous image CT-guided technique, as described by Cornelis [17], with pain relief obtained prevalently in the first month after the procedure and neurological improvement in the third month of follow-up [18]. The study’s data analysis revealed that only 21 out of 40 patients were evaluated at the three-month follow-up, which dampens the enthusiasm regarding the described results [18].

ECT for spine metastases presents effective outcomes but several inherent risks and challenges. Firstly, the proximity of the spinal cord and nerve roots to metastatic lesions increases the potential for neurological damage during the procedure [29]. This risk necessitates careful planning and precise electrode placement to minimize the likelihood of adverse events. A transpedicular ECT procedure combined with vertebral screw fixation in selected patients with tumor volume contained within the vertebral body minimizes the risk for neural damage [16,17,18]. There are important limits in this technique such as the position and angles of electrodes in pedicles and the difficulty of a follow-up examination of the treated lesion with standard imaging techniques due to pedicle screws [16]. A recent study confirmed that the deflection of needles alters the distribution of the electric field, reducing the efficacy of ECT [30]. This aspect could be overcome by the introduction of long-needle variable electrode geometry [31]. Secondly, spine metastases often involve critical structures such as vertebral bodies and the surrounding vasculature. ECT in this context requires meticulous attention to avoid injury to these structures, which could lead to consequences such as spinal cord compression or vascular compromise. Additionally, the efficacy of ECT may be limited in cases where metastases are located in regions of the spine that are difficult to access or where electrode placement is challenging. This may necessitate alternative treatment approaches or a combination of therapies to achieve optimal outcomes while mitigating risks. Close monitoring and follow-up are essential to detect early recurrence or disease progression and adjust treatment strategies accordingly. Overall, while ECT can be a valuable treatment option for spine metastases, its implementation requires careful consideration of the specific risks and challenges associated with this anatomical location, as well as a multidisciplinary approach involving oncologists, neurosurgeons, and interventional radiologists to ensure patient safety and treatment efficacy.

## 6. Conclusions

According to the literature review and our experience, ECT enhances the toxicity of bleomycin administrated systematically, changing the permeability of membrane cells. This effect is possible due to the modification of membrane cell polarization induced by electric pulses delivered near the tumor site with several techniques. ECT does not modify the overall survival of oncologic patients but is an alternative option for the treatment of spine metastases. Pain is a paramount issue in bone metastatic patients, and the findings of all studies in this review and our experience concur on the capability of ECT to improve neurological conditions and achieve pain relief.

Indeed, ECT is a safe, feasible, and minimally invasive rescue option for oncological patients with spine metastases and not an alternative to surgical resection; it has been shown to improve pain and neurological symptoms in both the short term and long term.

## Figures and Tables

**Table 1:** Results of literature review.

Ref. (Years)	Type Study	N. Pt	Type of Primary Tumor	Spine Level	ECT Electrode Placement	Outcome (*)
[12] (2015)	Case report	1	Melanoma	Lumbar	Surgical procedure: Hemilaminectomy	Pain relief Good MRI response
[16] (2018)	Case reports	2	N/A	Lumbar Thoracic	Minimally invasive: Transpedicular Approach	N/A
[17] (2019)	Case reports	2	Breast carcinoma; Lung carcinoma	Lumbar	Percutaneous CT guided (pedicles\vertebral body)	Pain relief Good MRI response
[18] (2023)	Case series	40	Thyroid carcinoma; Lung carcinoma; Sarcoma; Others (*)	Cervical Lumbar Thoracic	Percutaneous CT guided (pedicles\vertebral body)	Pain relief Neurological improvement Good MRI response
Present study	Case series	3	Myxofibrosarcoma; Squamous cell carcinoma of skin; leiomyosarcoma	Lumbar	Transpedicular approach during surgical procedure	Pain relief and neurological improvement **

(*) See Discussion section for more details. ** One patient died of disease 4 months after treatment and another one after 3 months due to renal failure.

## Data Availability

Manuscript data are embedded in the text and fully available upon specific request.
